# Integration of Vertical Graphene Onto a Tunnelling Cathode for Digital X‐Ray Imaging

**DOI:** 10.1002/advs.202403721

**Published:** 2024-08-15

**Authors:** Sahng‐Kyoon Jerng, Eunju Hong, Giwon Lee, Byungkee Lee, Jae Ho Jeon, Jinah Kim, Seung‐Hyun Chun

**Affiliations:** ^1^ Department of Physics Sejong University Seoul 05006 South Korea; ^2^ Digital X‐ray task Artificial Intelligence Lab LG Electronics Seoul 07796 South Korea

**Keywords:** ozone treatments, tunnelling cathodes, vertical graphene, X‐ray generation

## Abstract

As an alternative to thermionic X‐ray generators, cold‐cathode X‐ray tubes are being developed for portable and multichannel tomography. Field emission propagating from needle structures such as carbon nanotubes or Si tips currently dominates related research and development, but various obstacles prevent the widespread of this technology. An old but simple electron emission design is the planar tunnelling cathode using a metal–oxide–semiconductor (MOS) structure, which achieves narrow beam dispersion and low supplying voltage. Directly grown vertical graphene (VG) is employed as the gate electrode of MOS and tests its potential as a new emission source. The emission efficiency of the device is initially ≈1% because of unavoidable fabrication damage during the patterning processes; it drastically improves to >40% after ozone treatment. The resulting emission current obeys the Fowler–Nordheim tunnelling model, and the enhanced emission is attributed to the effective gate thickness reduction by ozone treatment. As a proof‐of‐concept experiment, a clustered array of 2140 cells is integrated into a system that provides mA‐class emission current for X‐ray generation. With pulsed digital excitations, X‐ray imaging of a chest phantom, demonstrating the feasibility of using a VG MOS electron emission source as a new and innovative X‐ray generator is realized.

## Graphical abstract

Vertical graphene is integrated onto a tunnelling cathode device, and the effective electrode thickness is reduced by ozone treatments for highly efficient electron emission. The demonstration of digital X‐ray imaging implies that compact X‐ray machines can be developed using a combination of graphene and silicon technology.

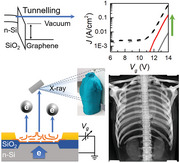

## 1 Introduction

From health diagnosis to drug detection, the use of X‐rays has become widespread, driving the expansion of many related industries. While the detection of X‐rays has evolved almost completely from the use of analogue films to digital data, the generation of X‐rays still depends heavily on traditional thermionic X‐ray tubes operated by heated filaments or hot cathodes. As the need for small, portable generators is increasing, new types of X‐ray generators are needed. The most successful candidates thus far seem to be field emission cathodes using carbon nanotubes (CNTs), which are already on the market and in use for portable X‐ray systems.^[^ ^1^, ^2^, ^3^ ^]^ However, the resulting needle structure subject to a high operating voltage suffers from brittleness upon exposure to the high local electric fields. Moreover, high vacuum conditions are required because the adsorption of gas molecules causes undesired effects. Si tip emitters also suffer from similar problems.

**Figure 1:**
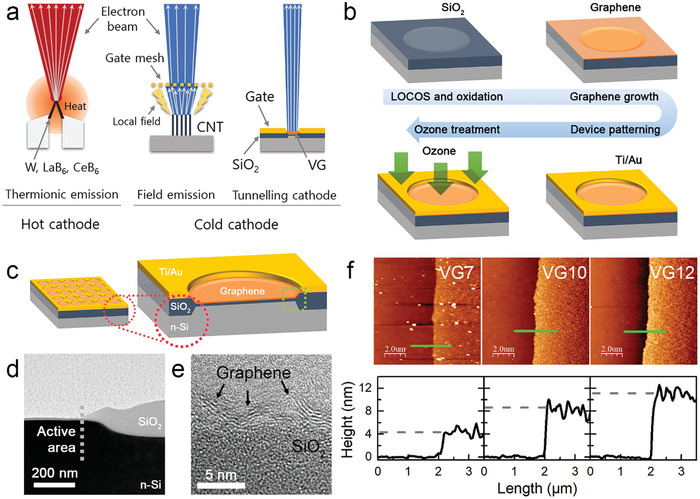
Vertical graphene‐gated tunnelling cathodes. a) Schematic illustrations of different types of electron emission devices. b) Schematics of device fabrication processes. c) Illustrations of an array of 25 cells and a single‐cell structure. d) Cross‐sectional TEM image at the green square in (c). e) HR‐TEM image of VG on SiO_2_. f) AFM images and line profiles for a nominal thickness of VG grown for 7, 10, and 12 min (labeled as VG7, VG10, and VG12).

An alternative and simpler electron emission design is to use planar metal–oxide–semiconductor (MOS) junctions. While field emission devices force electrons out of the cathode by a strong local electric field, a planar tunnelling cathode utilizes a MOS structure where electron tunnelling processes from a semiconductor cathode to a metal electrode occur. Once the tunnelling electrons possess higher kinetic energies than the work function of the electrode, they can escape to the vacuum and reach the anode. MOS junctions thus offer many advantages. The emission itself requires a low voltage of ≈10 V. Furthermore, the tunnelling cathodes have the advantages of vacuum‐independent operation, narrow beam divergence, fast‐switching, and beam modulation, compared to field emission devices^[^ ^4^ ^]^ (**Figure** **1**a). Despite the simplicity and advantages of thin‐film technology, MOS junctions have not been successful at producing electron emission because of their low efficiency (less than 1%). This low efficiency is mostly attributed to inelastic scattering at the electrodes.

The use of graphene as the electrode material may overcome the limitation. Recently, a series of studies reported highly efficient MOS structures utilizing few‐layer graphene films.^[^ ^5^, ^6^, ^7^, ^8^, ^9^ ^]^ Although the group has not yet reported a practical demonstration, the results are quite promising. The electron energy after tunnelling was far above the work function of graphene, which led to a high emission efficiency. They also demonstrated a large current from 380 arrays.^[^ ^5^ ^]^ Therefore, graphene and related 2D materials could be utilized for next‐generation cold‐cathode X‐ray generators.

We have observed that ultrathin vertical graphene (VG) structures have superior properties to flat graphene and are robust enough to survive the tough processes of device fabrication and integration. VG served as a robust support material for both acidic and alkaline conditions.^[^ ^10^ ^]^ These materials also exhibited remarkable durability under repeated strains.^[^ ^11^ ^]^ Importantly, a clear preference for free‐standing edges of graphene over flat surfaces was reported in a position‐dependent field electron emission study.^[^ ^12^ ^]^ However, previous studies of VG for electron emission employed several hundred‐nm‐thick VGs and measured vacuum emission in a diode geometry only to compare the performance with that of CNTs.^[^ ^13^ ^]^ Here, to apply the VG for MOS‐type X‐ray generation, we employed much thinner VG structures. Initially, we grew 4–11 nm thick VGs, and after device fabrication, we applied ozone treatment to restore the surface morphology and further decrease the effective thickness of the VG electrode. This ozone treatment increased the emission efficiency, and the highest efficiency reached ≈40% for the thinnest sample. To demonstrate the feasibility of the VG X‐ray generator, a lower‐efficiency sample with better durability was used to match industrial demands. A clustered array of 2140 cells was integrated to function as an mA‐class electron source, which in turn generated pulses in the X‐ray and successfully imaged a chest phantom. We propose that our VG X‐ray generator represents a next‐generation, planar MOS‐type tunnelling cathode X‐ray system for inexpensive and mobile X‐ray imaging.

## 2 Results and Discussion

To integrate VG into an electron emission device, we combined an advanced microfabrication technique of silicon and a catalyst‐free, plasma‐enhanced chemical vapor deposition (PECVD) graphene growth technique. Ozone, generated by a UV lamp, was exposed to the VG MOS devices for 5–30 min (Figure 1b). Further details of the fabrication processes are provided in Figure S1 (Supporting Information). Figure 1c shows a schematic illustration of the device comprising a 5 × 5 array of electron emission cells. An enlarged cross‐sectional illustration of a single cell and a TEM image of a 10 nm‐thick SiO_2_ tunnel barrier connected to a thicker field oxide are presented in Figure 1d. The local oxidation of silicon (LOCOS) process was used to isolate each cell and to avoid electron tunnelling through metal contacts by forming a field oxide in selected areas on a silicon wafer. VG growth on SiO_2_ started from the horizontal growth of buffered graphene layers a few nanometers thick. This buffered graphene was different from the directly grown flat graphene because the lateral grain sizes were only a few nanometers and the thicknesses were not uniform.^[^ ^14^ ^]^ The defective edges of small grains played a role as random growth seeds, giving rise to vertical graphene overlayers, as described in our previous reports (see also Figure 1e).^[^ ^11^, ^15^ ^]^

**Figure 2:**
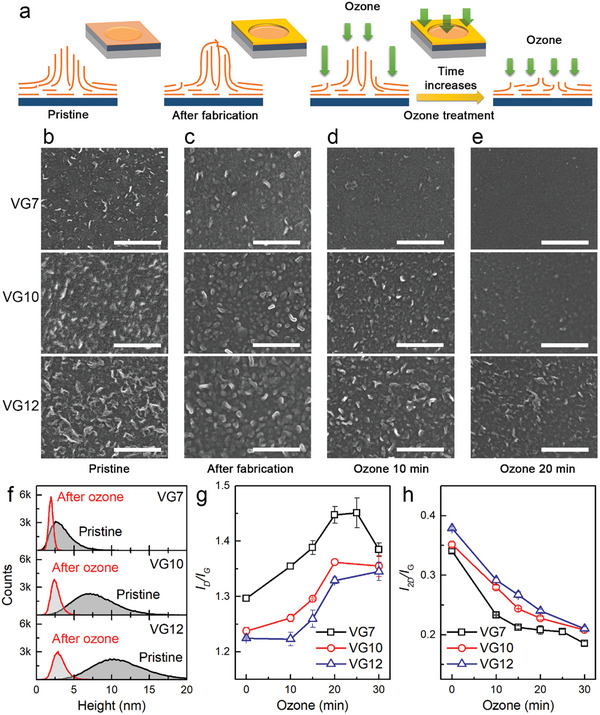
Influence of ozone treatment on the VG structure. a) Schematic illustrations of VG structure changes. b–e) FESEM images of VG surface changes caused by fabrication and ozone treatment (scale bar = 200 nm). f) Histograms of morphology profile changes measured by AFM before/after long ozone treatment (20 min) show that the height becomes smaller with a much narrower distribution after a long time ozone treatment. g,h) Normalized Raman intensity ratio of D and 2D peaks to G peak.

In this study, samples were labelled based on VG growth time on a minute scale, and the approximate thicknesses of VG7, VG10, and VG12 were 4.3, 8.4, and 11.1 nm, respectively (see Figure 1f for AFM images and line profiles). Above the buffered graphene, the VG nanowalls protruded vertically (**Figure** **2**a). For such a short VG, the surface morphology changed significantly as the growth time increased, as shown in Figure 2b. The distribution of VG heights and their dependence on the growth time will be discussed in more detail below. We first focused on the changes in morphology, driven by the fabrication process and ozone treatment. As shown by the wavy patterns in Figure 2c, the clear nanowall edges of graphene became blunt during the device patterning process. It has previously been shown that the VG can withstand photolithography and lift‐off processes. However, the open graphene edges could be chemically modified. Closing of the VG ends, as proposed in Figure 2a, was one of the possible approaches. Certain chemical residues could be attached to the VG ends. Regardless of the origin of these wavy patterns, the as‐prepared devices showed negligible emission efficiencies. To increase the electron emission, we attempted UV‐ozone treatments, expecting the exposure of highly reactive graphene edges and a decrease in the overall electrode thickness simultaneously, as illustrated in the schematics of Figure 2a. Indeed, the ozone treatment progressively changed the morphology as the ozone exposure time increased (Figure 2d,e). The sharp VG edges were recovered, and the resulting morphology followed that of the thinner samples regardless of the growth time. The evolution of the surface morphology was observed more clearly when we repeated the same procedure using the bare VG samples rather than the patterned samples. The SEM images in Figure S2 (Supporting Information) show that the ozone treatment progressively etched the VGs, decreasing their length. Interestingly, the morphologies of the as‐grown VG7, ozone 10 min VG10, and ozone 20 min VG12 meteorites are similar. Therefore, the thick VG can be transformed to thinner VG samples by ozone treatment. The height distribution also changed in response to the ozone treatment. The broad height distribution changed to a shorter VG with a narrower distribution, as determined by AFM (Figure 2f; S3, Supporting Information). However, the histogram of ozone 20 min VG12 was similar to that of as‐grown VG7, indicating that the buffered graphene was also etched and that the average thickness should be the same. Additional evidence supporting this conclusion is observed in the Raman spectra, as presented in Figure S4 (Supporting Information). The influence of VG caused by ozone had an increasing D peak intensity (*I*_D_) and a decreasing 2D peak intensity (*I*_2D_) compared to the G peak intensity (*I*_G_), indicating an increase in roughness or defective edges (Figure 2g,h). Again, *I*_D_/*I*_G_ are similar for 20 min of VG12 and as‐grown VG7. Therefore, ozone treatment is an effective method for restoring chemically modified VGs after fabrication and controlling the effective thickness during post‐processing.

**Figure 3:**
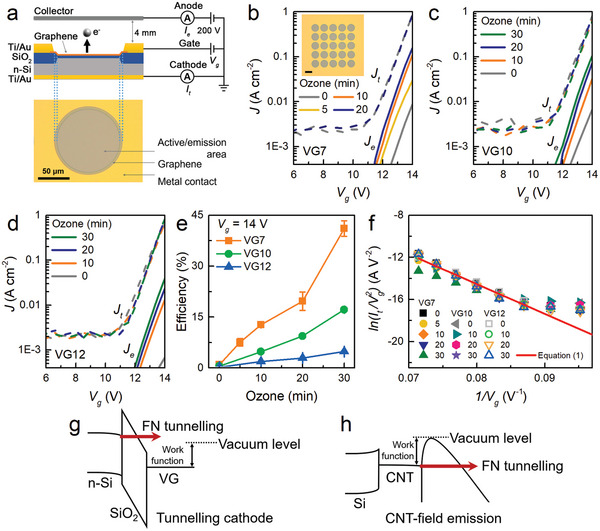
Electron emission properties for VG samples with different ozone treatment times. a) Schematic illustration of the electron emission measurement setup and optical image of a single cell. b) *J–V* curves of the VG7 samples for different ozone treatment times. (Inset) Optical image of a 5 × 5 array device (scale bar = 100 µm). c,d) *J–V* curves of the VG10 and VG12 samples. e) Emission efficiency dependences of the VG7, VG10, and VG12 samples on the ozone treatment time. f) Total current FN plots for all the samples presented. g) Illustration of the tunnelling cathode emission mechanism and h) CNT‐based field emission mechanism.

To determine the electron emission properties of MOS devices employing VG as the gate electrode, a standard three‐terminal method was used. The n‐Si substrate was grounded and used as the cathode. A positive gate voltage was applied from a voltage source to the VG layer via Ti/Au metal contacts. Some of the tunnelling electrons through the SiO_2_ barrier in the active area were emitted from the VG and reached the anode, a stainless‐steel plate collector biased at 200 V; a schematic of the measurement setup and a 100 µm‐diameter single‐cell image are provided in **Figure** **3**a. The total current (*I_t_*) measured from the cathode and the emission current (*I_e_*) from the anode were converted to current densities *J_t_* and *J_e_*, respectively, using the total emission area of the array (the inset of Figure 3b). Figure 3b shows the electrical characteristics of MOS junctions with the VG7 series. While the total current density (*J_t_*) did not change before or after ozone treatment, the emission current density increased significantly as the ozone treatment time increased. The electron emission turn‐on voltage also decreased to ≈11.5 V, where the cathode current density started to increase exponentially. Similar behaviors were also observed for the VG10 and VG12 series (Figure 3c,d).

For the tunnelling cathode electron emission devices, the figure of merit is the emission efficiency (*η*), defined by the ratio of the emission current to the cathode current at a specified voltage. It is an important factor not only for X‐ray generation but also for general vacuum microelectronics such as flat panel displays. High emission efficiency results in low power consumption per emission current, which helps reduce heat and prolong the lifetime. As summarized in Figure 3e, *η* at *V*_g _= 14 V was greater for shorter VG growth and longer ozone treatment. The maximum efficiency reached ≈40%, far beyond the values reported for Au or Si gate MOS cathodes (<1%)^[^ ^4^, ^16^, ^17^ ^]^ and even greater than that reported for the latest flat graphene MOS junctions (32%).^[^ ^7^ ^]^

To understand the general features of our devices, we divided the entire emission process into two separate steps: electron tunnelling from the cathode to the VG gate through the insulator, and electron emission from the VG gate to the anode through vacuum. The first step is to send the electron through the classically forbidden barrier. If the barrier is thin enough, then the electron can tunnel directly even at low gate voltages. However, Figure 3b–d shows no sign of direct tunnelling, and the current density becomes significant only at large gate voltages. Direct tunnelling is known to be insignificant even for a 5 nm thick SiO_2_ barrier.^[^ ^18^ ^]^ Thus, for our 10 nm‐thick SiO_2_ film, a large gate voltage is needed to increase the potential difference at the SiO_2_ interfaces and to decrease the effective tunnelling barrier. If we neglect the accumulation layer, the tunnelling current should follow the Fowler–Nordheim (FN) equation:^[^ ^18^, ^19^, ^20^, ^21^, ^22^, ^23^, ^24^ ^]^

(1) $$I\;\propto \;V^{2}\exp\left(-\frac{bd\varphi^{3/2}}{V}\right)$$

where *d* is the barrier width, *φ* is the barrier height at the injection interface (≈3.25 eV),^[^ ^25^ ^]^ and *V* is the bias voltage. The FN constant $b\;=\;\;4\sqrt{2m_{e}}/3e\hbar =$6.83 × 10^9^ V eV^−3/2^ m^−1^ in the elementary FN equation should be reduced with the effective electron mass of SiO_2_, $m_{SiO_{2}}$. The emission data can be compared to the FN equation by checking the linearity of *ln(I/V^2^)* versus *1/V*, known as the FN plot. Figure 3f shows the FN plots for all the *I_t_–V_g_* curves. All the curves show good linearity and even lie together except for that from the 30 min ozone‐treated VG7 sample. The line in Figure 3f is from Equation (1) assuming $m_{SiO_{2}}=\;\;0.5m_{e}$.^[^ ^21^ ^]^ This confirms that the electron transfer from the cathode to the VG gate layer is governed by FN tunnelling and that all the devices are prepared reproducibly. The deviation of the 30 min ozone‐treated VG7 (Figure S5, Supporting Information), which exhibited the highest efficiency, can be explained by the large serial resistance reducing the effective gate voltage.

After FN tunnelling, the electrons spend some time in the conduction band of the oxide layer, leading to inelastic scattering with optical phonons.^[^ ^16^, ^26^ ^]^ Additional energy loss occurs when electrons travel through the gate electrode to reach the top surface. Finally, electrons with kinetic energies larger than the work function of the electrode can reach the anode. At large gate voltages for meaningful emission currents, the tunnelling barrier becomes very thin, and the electrons are thermally relaxed to exhibit symmetric distributions. However, previous studies with flat graphene showed that the devices behaved as hot electron transistors and the lowest energies were still larger than the work function of graphene.^[^ ^6^, ^27^, ^28^ ^]^ Thus, we expect the emission current to be proportional to the cathode current, as there is no cut‐off at the electrode vacuum level.^[^ ^29^ ^]^

At this point, it may be instructive to compare the emission mechanisms of tunnelling cathodes and field emission devices. The common feature is the FN tunnelling under a high electric field of ≈1000 V µm^−1^. For a tunnelling cathode, a gate voltage of ≈10 V is needed over 10 nm‐thick SiO_2_ between n‐Si and VG. For a CNT field emission device, for example, a nominal electric field of 1 V µm^−1^ is applied on the surface of the tips, where the geometrical enhancement makes the effective electric field strength to ≈1000 V µm^−1^, enabling FN tunnelling of electrons to the vacuum.^[^ ^30^ ^]^ Here, the tip diameter is an important parameter determining the field enhancement factor. In contrast, in our devices, the electric field at the VG surface is 0.05 V µm^−1^ nominally, and the enhancement factor is less than 40 at best.^[^ ^31^ ^]^ Therefore, the emission at the VG surface is due to hot electrons, not from another FN tunnelling (Figure 3g,h).

**Figure 4:**
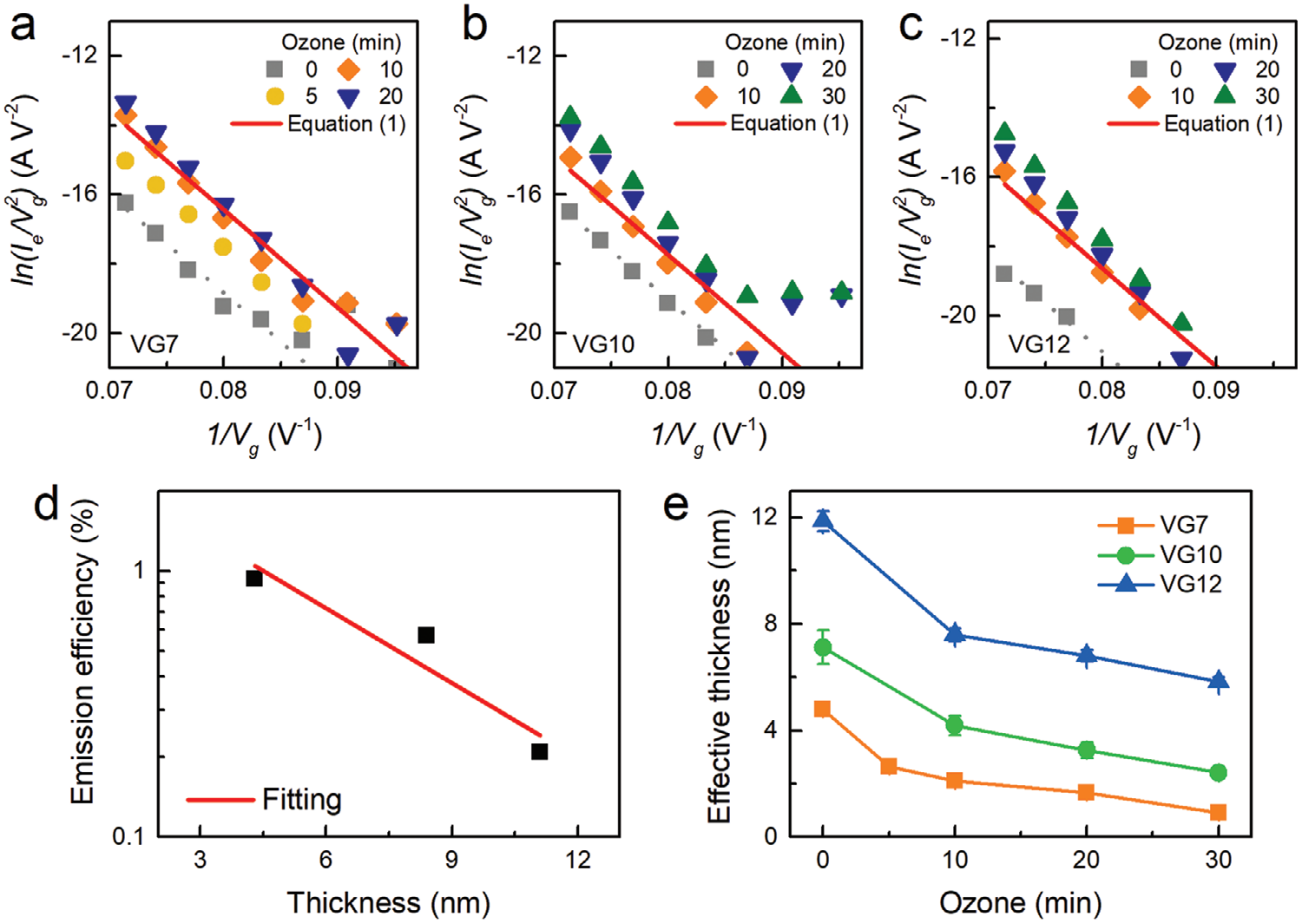
a–c) Emission current FN plots for the VG7, VG10, and VG12 series. The solid lines are from Equation (1) and have the same slope as in Figure 3f. d) Thickness versus emission efficiency plot. e) Estimated effective thickness as a function of ozone treatment time.

Indeed, **Figure** **4**a–c shows that the emission currents also exhibit good linearity in the FN plot and the slopes are more or less the same as that of the total current in Figure 3f or Equation (1). The effect of the VG gate is to shift the line, namely the amount of emission current. For all the thicknesses, the current strongly depends on the ozone treatment time. The longer the treatment time is, the greater the current and emission efficiency are. Overall, the increase in the emission current can be explained by the decrease in the electrode thickness, as the efficiency depends exponentially on the gate thickness *t*:^[^ ^4^, ^32^, ^33^ ^]^

(2) $$\eta \;\propto \;\exp\left(-\frac{t}{\lambda }\right)$$

where *λ* is the inelastic mean free path. From the variation in the efficiency of untreated devices, we estimated *λ* to be 4.6 nm (Figure 4d). It has been shown that *λ* depends on the electron energy and interestingly follows the universal curve.^[^ ^34^ ^]^ Our estimation of λ thus corresponds to a kinetic energy of 5.6 eV, which is reasonably between the work function of graphene (4.5 eV)^[^ ^35^ ^]^ and the applied gate voltage. With the initial thicknesses of the as‐grown VG and the emission efficiency, we estimate the effective thickness using Equation (2), and the results are shown in Figure 4e. The highest efficiency is observed when the effective thickness becomes a fraction of *λ*. The sample used in the demonstration of X‐ray images has an effective thickness of *λ*. This explains why the MOS employing a VG electrode outperforms other MOS structures. Previously reported MOS cathodes used a gate electrode made of Au, Al, or Pt. However, pinhole‐free thin films cannot be easily produced,^[^ ^32^ ^]^ and typical electrode thicknesses are multiples of *λ*.^[^ ^4^ ^]^ The poly‐Si gate showed much better efficiency,^[^ ^4^ ^]^ but the optimized thickness was still 5 times the *λ*.^[^ ^29^ ^]^ The minimum thickness was set because of the low lateral conductivity of poly‐Si. When the electrode thickness was a multiple of *λ*, the electrons experienced strong scattering, and the kinetic energies decreased close to the electrode vacuum level. Therefore, reducing the work function was necessary for higher emission efficiency.^[^ ^29^ ^]^ The advantages of VG include high conductivity and surface coverage even at a thickness of *λ*. Therefore, the electron kinetic energy was retained above the work function, leading to high emission efficiency at relatively low gate voltages.

An electron emission efficiency as high as 40% at *V*_g _= 14 V shows the potential of VG as a promising emitter. The value of 10 mA cm^−2^ for our 5 × 5 array corresponds to 20 µA, which is sufficient for electron microscopy applications where our VG tunnelling cathode devices can be applied.^[^ ^7^ ^]^ For X‐ray generation, however, the emission current should be increased to an industrial demand of more than 1 mA. However, we cannot simply increase the cell size and leave the number of arrays unchanged. The emission current density decreases as the unit emission area increases because the lateral electrode resistance reduces the effective gate voltage.^[^ ^5^ ^]^ A better technique is to increase the number of arrays, in which the LOCOS process plays a vital role. We treat the VG10 series with 10 min of ozone to achieve a better trade‐off between the emission efficiency and lateral resistance because device heating should be avoided because of diode current sinking in mA‐class emission devices.

**Figure 5:**
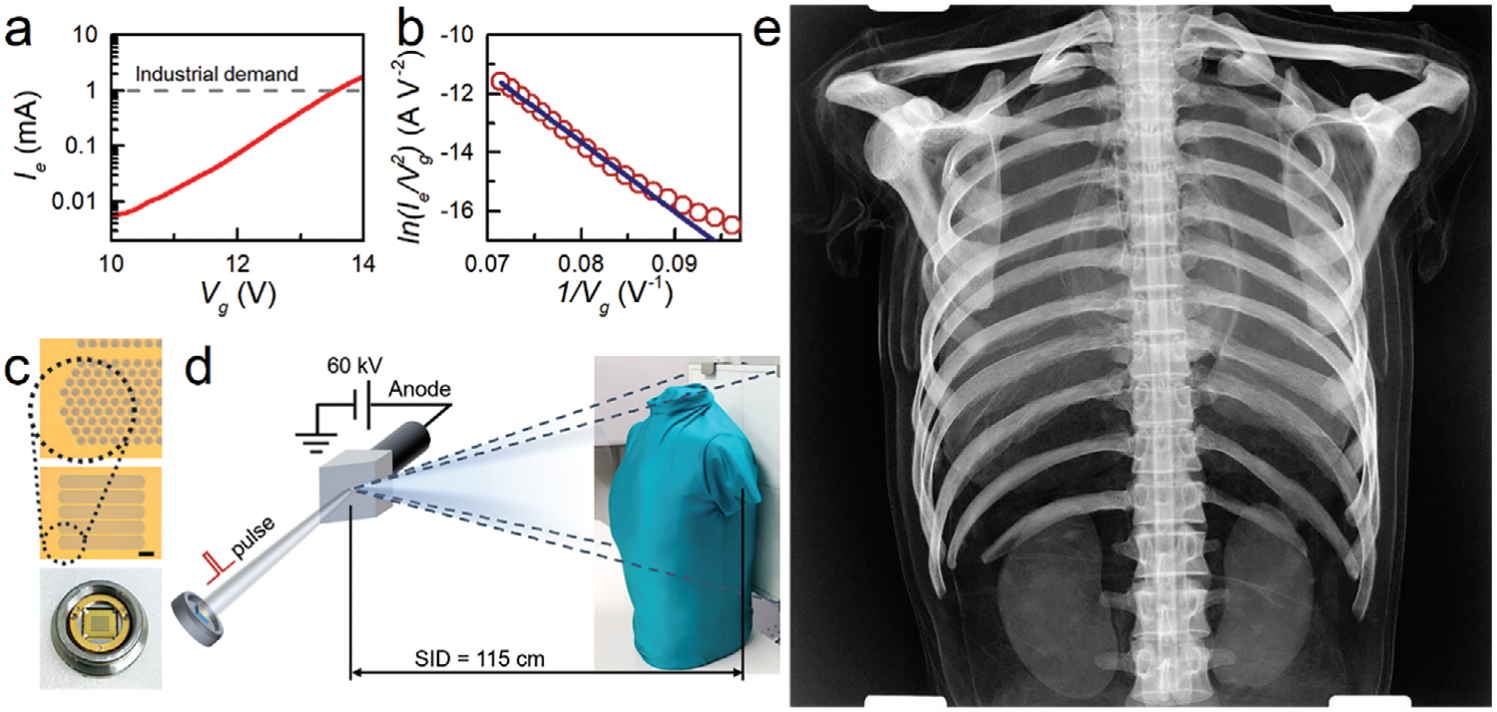
Digital X‐ray images of the chest phantom were acquired using a VG electron emission device. a) Emission current dependence on the gate voltage, b) the corresponding FN plot, and c) optical images (scale bar = 1 mm) and a photograph of the 2140 cell device. d) Schematic of X‐ray imaging of a chest phantom. e) Processed X‐ray image.

**Figure** **5**a,b shows the dependence of the emission current on the VG gate voltage for a total of 2140 cells consisting of 5 × 428 cells (Figure 5c). The emission current reached 1.8–2.2 mA at *V*_g _= 14 V, and the corresponding FN plot in Figure 5b confirmed that the practical device also followed the FN tunnelling mechanism. The electron emission cost (calculated from the input power divided by the emission current) and the emission current density of VG MOS junctions are comparable to those of conventional field emission devices^[^ ^36^, ^37^ ^]^ (see Supporting Information S6), raising the potential of VG X‐ray generators. As a proof‐of‐concept experiment, we performed digital X‐ray imaging in a custom vacuum chamber with a chest phantom. Figure 5d shows a schematic of our X‐ray system; the geometry of the X‐ray imaging system is provided in Supporting Information S7. The electrons emitted by the 100 ms pulse collide at the angled anode plate biased at 60 kV, and the generated X‐ray passes through the chest phantom and is recorded with a commercial 17 × 17 inch X‐ray detector panel. The source‐to‐image distance, defined by the distance between the center of the anode and the X‐ray detector panel, was set to 115 cm to simulate the real world. As shown in Figure 5e, the inner structures of the chest phantom were clearly scanned by electron emission from the VG‐cells. While the present radiation level is modest, it can be improved by further integration and tailoring of the insulating barrier, indicating the potential of using a VG tunnelling cathode device as a practical X‐ray generator.

**Figure 6:**
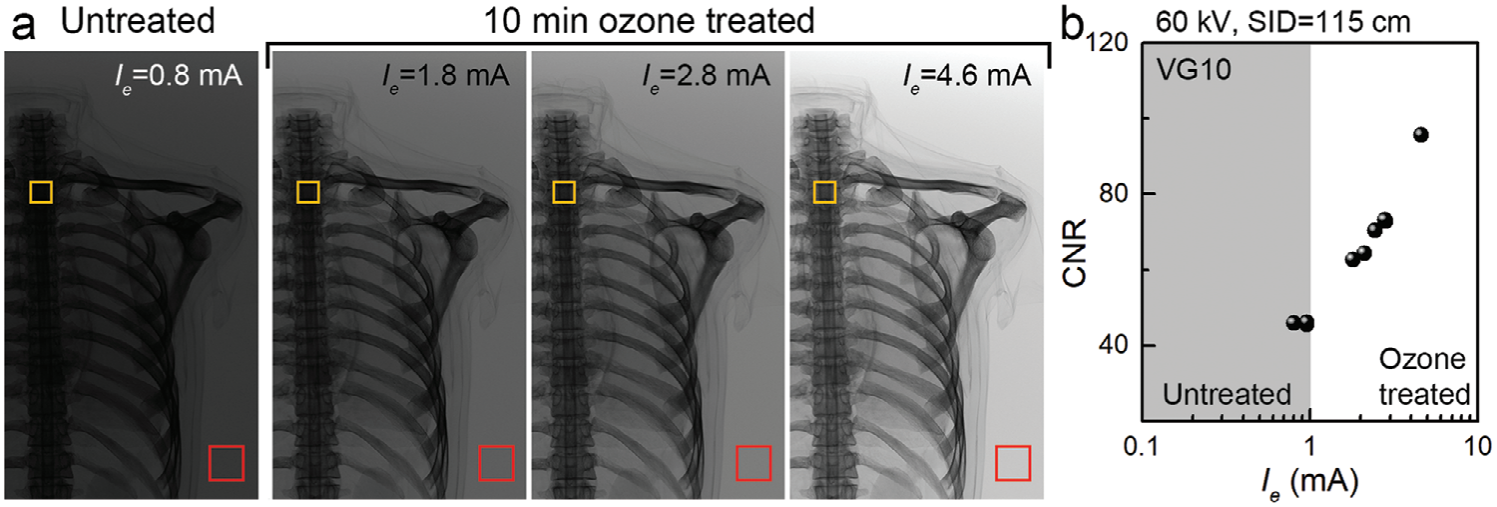
CNR analysis for the detected images. a) X‐ray images on the detector and ROI markers; orange boxes for ROI_o_ and red boxes for ROI_b_. b) Calculated CNR on *I_e_* changes from ozone‐treated and untreated VG devices.

The detector pixels record the X‐ray signal and return the brightness of the images. Without ozone treatment, *I*_e_ cannot exceed the industrial demand current level and the X‐ray images show low brightness as shown in **Figure** **6**a. In comparison, high brightness obtained from the ozone‐exposed VG results in an excellent contrast. To evaluate the X‐ray image quantitatively, we determined the performance in terms of contrast‐to‐noise ratio (CNR), an important factor for the viability of clinical applications. CNR is calculated by the following equation:^[^ ^38^, ^39^ ^]^

(3) $$\mathrm{CNR}\;=\left|PV_{o}-PV_{b}\right|\;/\sigma_{b}$$

where *PV*_o_ and *PV*_b_ are mean pixel values measured at the region‐of‐interest (ROI) for the object area (ROI_o_) and background area (ROI_b_), and *σ*_b_ is the background standard deviation. Figure 6b shows the CNR values for VG with/without ozone‐treated devices. The background area (marked by the red box, ROI_b_) and the spine (marked by the orange box, ROI_o_) were selected to calculate CNR. The CNR is limited to 46 for untreated VG, and it improves to 96 for an ozone‐treated VG device because of the enhanced emission current. To increase the accuracy of lesion diagnosis, a high CNR generated by incidence X‐ray is inevitably demanded and the CNR value close to 100 from an ozone‐treated VG device shows the potential of tunnelling cathodes in the medical field.

A significant bottleneck for application is the lifetime at high current levels. The oxide layer is still too thick to avoid charge trap generations within the layer. Oxide reliability is known to strongly increase for thinner oxides, less than 3 nm in thickness in the case of SiO_2_.^[^ ^18^ ^]^ At this thickness, direct tunnelling current can be observed and utilized for electron emission. The oxidation method might have to be changed from thermal to CVD deposition along with an advanced isolation process. As direct growth of VG is possible on most oxides, the adoption of high‐k oxide can be another direction for extending the device lifetime.

## 3 Conclusion

In this study, we demonstrate that the VG‐integrated MOS exhibits electron emission properties similar to or better than those of graphene MOS. Moreover, we show that the emission efficiency can be controlled by a post‐processing method: UV‐ozone treatment. The gate voltage dependence of the emission current is attributed to FN tunnelling through the SiO_2_ barrier and inelastic scattering at the electrode. We attribute the effect of ozone treatment to a reduction in the effective VG thickness, equal to or less than the inelastic mean free path, which reasonably explains the change in emission efficiency. To demonstrate practical feasibility, we fabricated an array of 2140 cells to attain an industrially demanding emission current level (>1 mA). We successfully obtained digital X‐ray images, opening the door to innovative X‐ray generators utilizing planar MOS junctions. We anticipate that our work will generate interest in tunnelling cathode devices with 2D materials. The demonstration of X‐ray imaging is evidence that MOS structures could also be applied in various electron emission devices. When electron emission is properly converted to light generation, we may end up with precise control of photon generation at high operation speed.

## 4 Experimental Section

### Device Fabrication Process

An n‐type Si (100) substrate with a resistivity of 1–10 Ω cm was used in this study. A field oxide of a 200 nm‐thick SiO_2_ formed through the LOCOS process with BHF and SC1, known as RCA cleaning. A tunnel barrier layer with a thickness of 10 nm SiO_2_ was grown by thermal oxidation at 800 °C. As a gate electrode, VG was directly grown by PECVD at 750 °C for VG7 (7 min), VG10 (10 min), and VG12 (12 min) using a mixture of gases (10 sccm of CH_4_ and 10 sccm of H_2_) discharged at a radio frequency power of 50 W. Further details of graphene growth could be found elsewhere.^[^ ^10^, ^11^, ^15^, ^40^, ^41^, ^42^, ^43^ ^]^ The top and bottom electrodes, consisting of 30 nm‐thick Ti and 200 nm‐thick Au, were formed by a lift‐off process with e‐beam evaporator deposition. Ozone was generated by UV light at wavelengths of 184.9 and 253.7 nm for a ratio of 2:8.

### Characterization of Graphene

Images of the graphene surfaces of the as‐grown samples (SU8010, Hitachi, Japan) and fabricated samples (GeminiSEM 300, ZEISS, Germany) were obtained via FESEM using an accelerating voltage of 7 kV. Cross‐sectional images were obtained via HR‐TEM (TitanTM 80–300) operated at 200 keV. Raman spectra were recorded by a micro‐Raman spectroscopy system (inVia, Renishaw, UK) with a 514.5 nm laser source. The surface morphology and thickness of the graphene layer were evaluated via an atomic force microscopy (AFM) instrument (n‐Tracer system, Nano Focus, Korea) in noncontact mode.

### Electron Emission Property Measurements

The electron emission measurements were carried out using a vacuum chamber at 1 × 10^−6^ Torr. For *I–V* characterizations, Keithley 4200A‐SCS parameter analysers were employed to acquire the tunnelling current and the emission current from the cathode and the anode, respectively, as a function of the gate voltage applied to the VG layer, while the collector was biased at 200 V.

### X‐Ray Imaging

The sample was loaded on a custom X‐ray system dedicated to obtaining X‐ray images, employing a vacuum chamber maintained at 1 × 10^−7^ Torr. The gate was biased by a 100 ms pulse using a Keithley 2636B sourcemeter. The distance between the sample and the tungsten anode was 80 mm. A commercial X‐ray detector panel (17HK700G‐W, LG Electronics, Korea) was used to record X‐ray images. The geometry and photograph of the X‐ray imaging system are presented in Figure S7 (Supporting Information).

## Conflict of Interest

E.H., G.L., B.L., and J.K. have filed a patent on the technology in this paper. The other authors declare no conflict of interest.

## Supporting information

Supporting Information

## Data Availability

The data that support the findings of this study are available from the corresponding author upon reasonable request.
