# Supplementary material for: Integration of Vertical Graphene Onto a Tunnelling Cathode for Digital X‐Ray Imaging
Source: Adv Sci (Weinh). 2024 Aug 15;11(39):2403721. doi: 10.1002/advs.202403721 (PMC11497061; doi:10.1002/advs.202403721)
Supplement: Supplementary file 1 — Supporting Information [file ADVS-11-2403721-s001.docx]

Supporting Information

Integration of vertical graphene onto a tunnelling cathode for digital X-ray imaging

Sahng-Kyoon Jerng,^*^ Eunju Hong, Giwon Lee, Byungkee Lee, Jae Ho Jeon, Jinah Kim,^*^ and Seung-Hyun Chun,^*^

Table of contents:

S1. Device fabrication

S2. Ozone treatment for bare VG

S3. AFM images of long time ozone exposed VG

S4. Raman spectroscopy analysis

S5. *J-V* characteristics for 30 min ozone treated VG7

S6. Electron emission cost

S7. X-ray imaging system

S1. Device fabrication

Figure S1 shows the overall fabrication process. Using a local oxidation of silicon (LOCOS) process with 160 nm-thick Si_3_N_4_, a field oxide was formed on selective area to separate the emission cells. The emission area was etched by buffered oxide etch 7:1 solution prior to form a 10 nm-thick SiO_2_ layer by the thermal oxidation. Before/after the LOCOS process, the surface was cleaned by a standard clean (called SC-1) to avoid unintentional contaminations. For the graphene growth, the substrate was cleaned by ex-situ ozone exposure and O_2_ plasma (40 sccm, 50 W) in a high vacuum chamber. Vertical graphene (VG) was directly grown on SiO_2_ surface by plasma enhanced chemical vapor deposition in 10 mTorr maintained by a throttle gate valve.^[1–5]^ As-grown VG layer was patterned by a photolithography and O_2_ plasma etching. Metal electrodes were deposited by e-beam evaporation, using lift-off layer method to pattern a top electrode. The ozone treatment was performed before electron emission measurements.


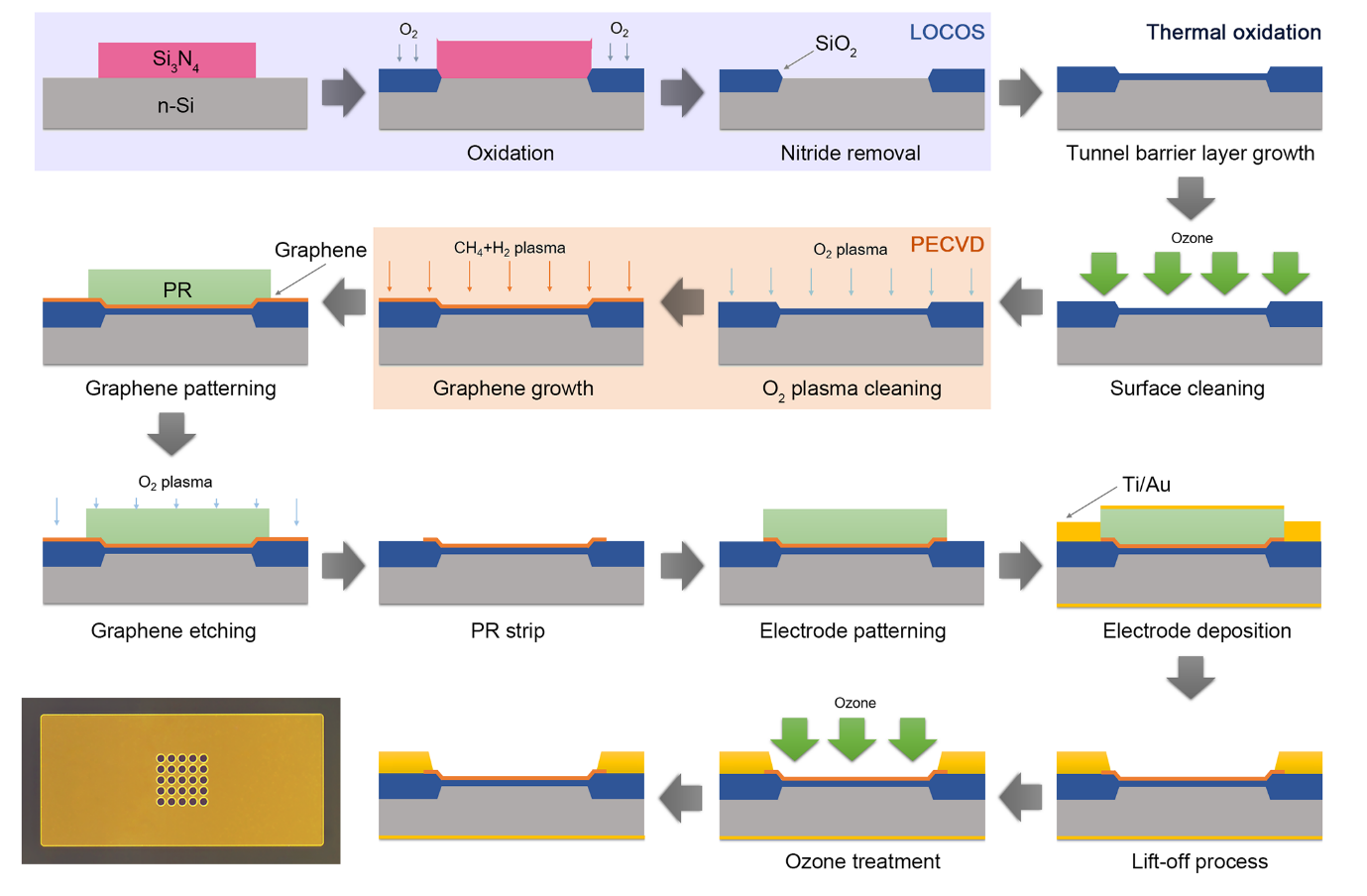


**Figure S1.** Illustration of detailed device fabrication steps for VG electron emission device.

S2. Ozone treatment for bare VG

Figure S2 shows the morphology change by ozone treatment for bare VG samples. Due to the absence of patterning process, the ozone exposed VG shows progressive changes, showing that ozone reacts with the graphene edges to sharpen VG.


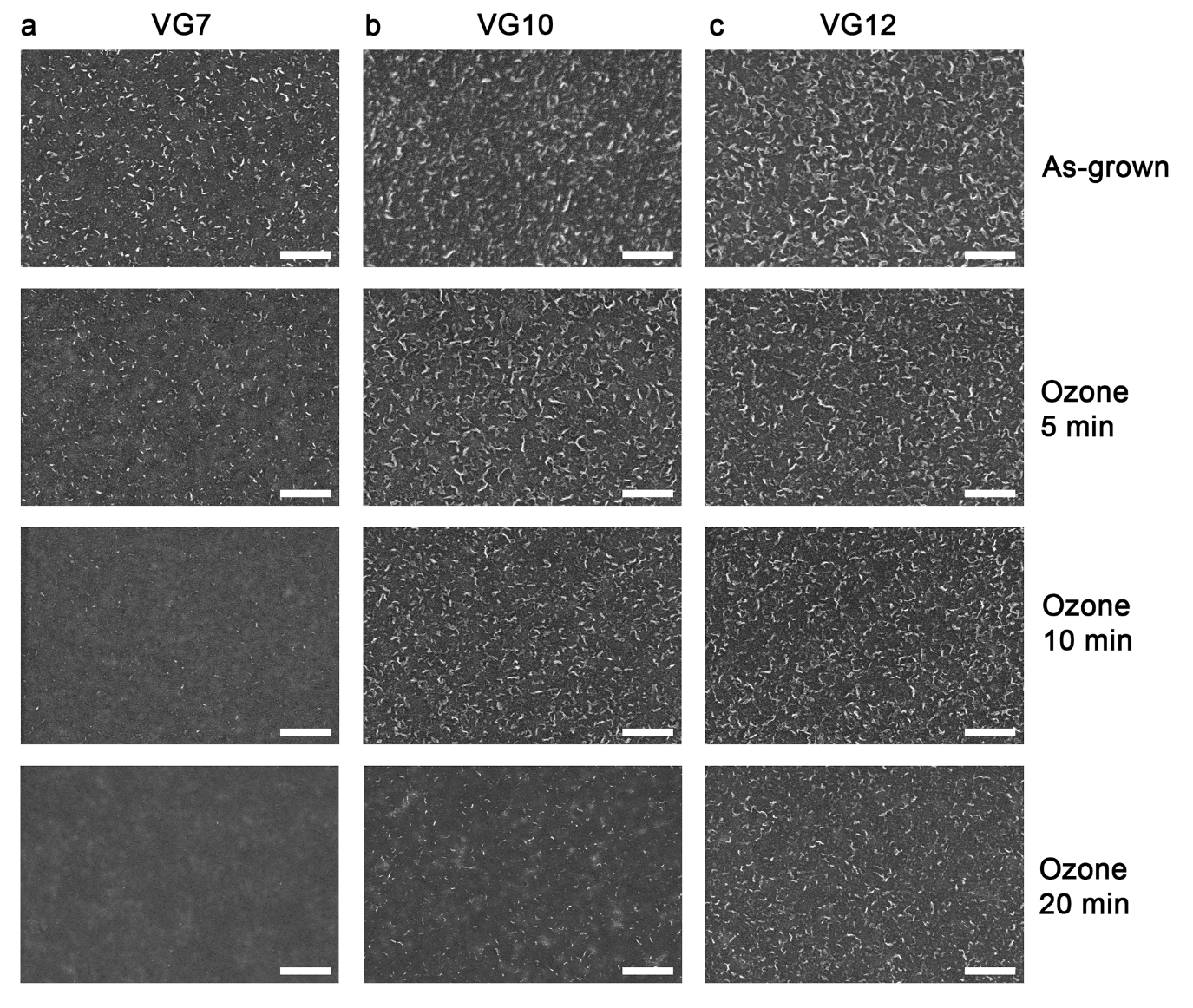


**Figure S2.** FESEM images of a) VG7, b) VG10, and c) VG12 (scale-bar = 200 nm) without device fabrication. Due to the absence of VG collapsing by fabrication, VG structures are rapidly sharpened/shortened by ozone treatment.

S3. AFM images of long time ozone exposed VG

While the FESEM images of ozone treated VG indicate the etching effect, Figure S3 shows that the exposed buffered graphene layer still possesses numerous/short spikes of VG. Histograms of morphology profile are available in the main text (Figure 2f).


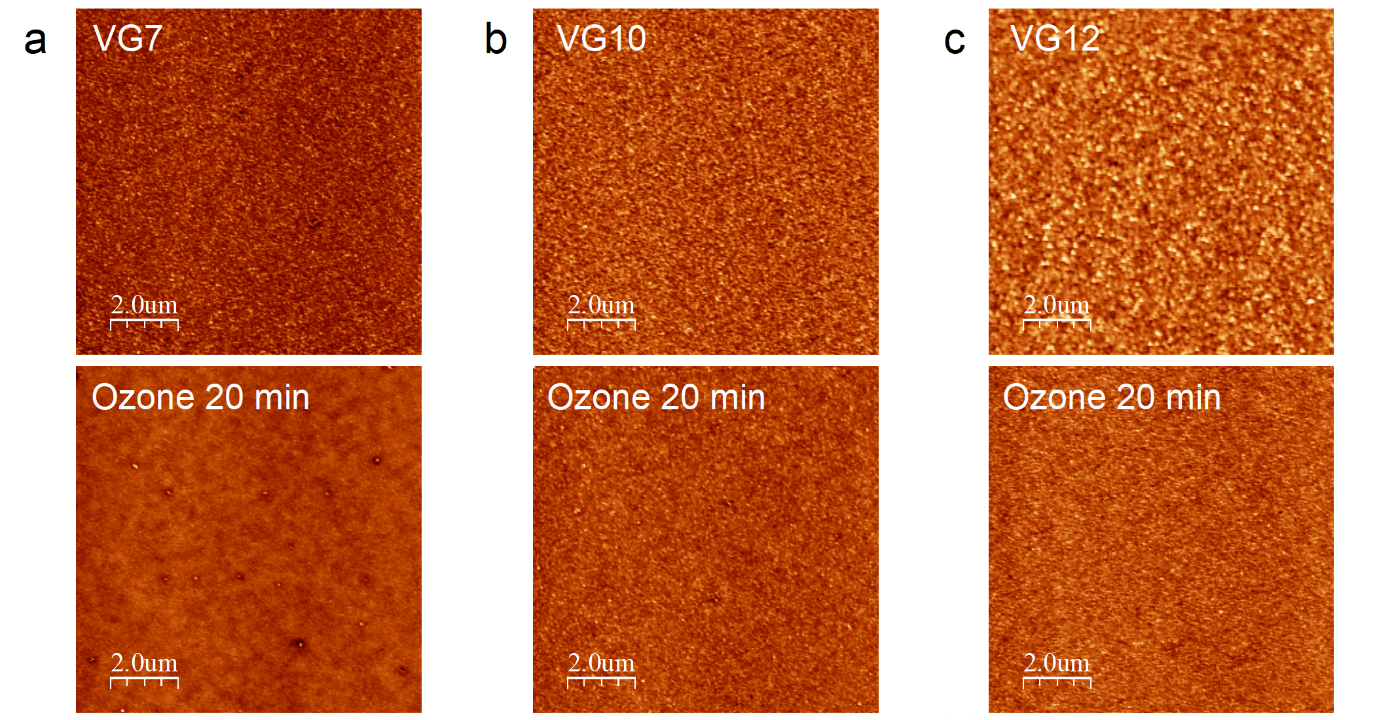


**Figure S3.** Morphology changes of a) VG7, b) VG10, and c) VG12 after long ozone treatment of as-grown samples.

S4. Raman spectroscopy analysis

Figure S4 presents the Raman spectra of VG as the ozone treatment time increases; peaks are normalized by using the G peak height (~ 1590 cm^-1^). The graphene influenced by ozone becomes defective as indicated by the increasing D peak (~ 1350 cm^-1^). The decrease of 2D peak (~ 2700 cm^-1^) also confirms the previous observation (S3) that the buffered graphene has short spikes. The analysis for the ratio of peaks are available in the main text (Figure 2g and 2h).


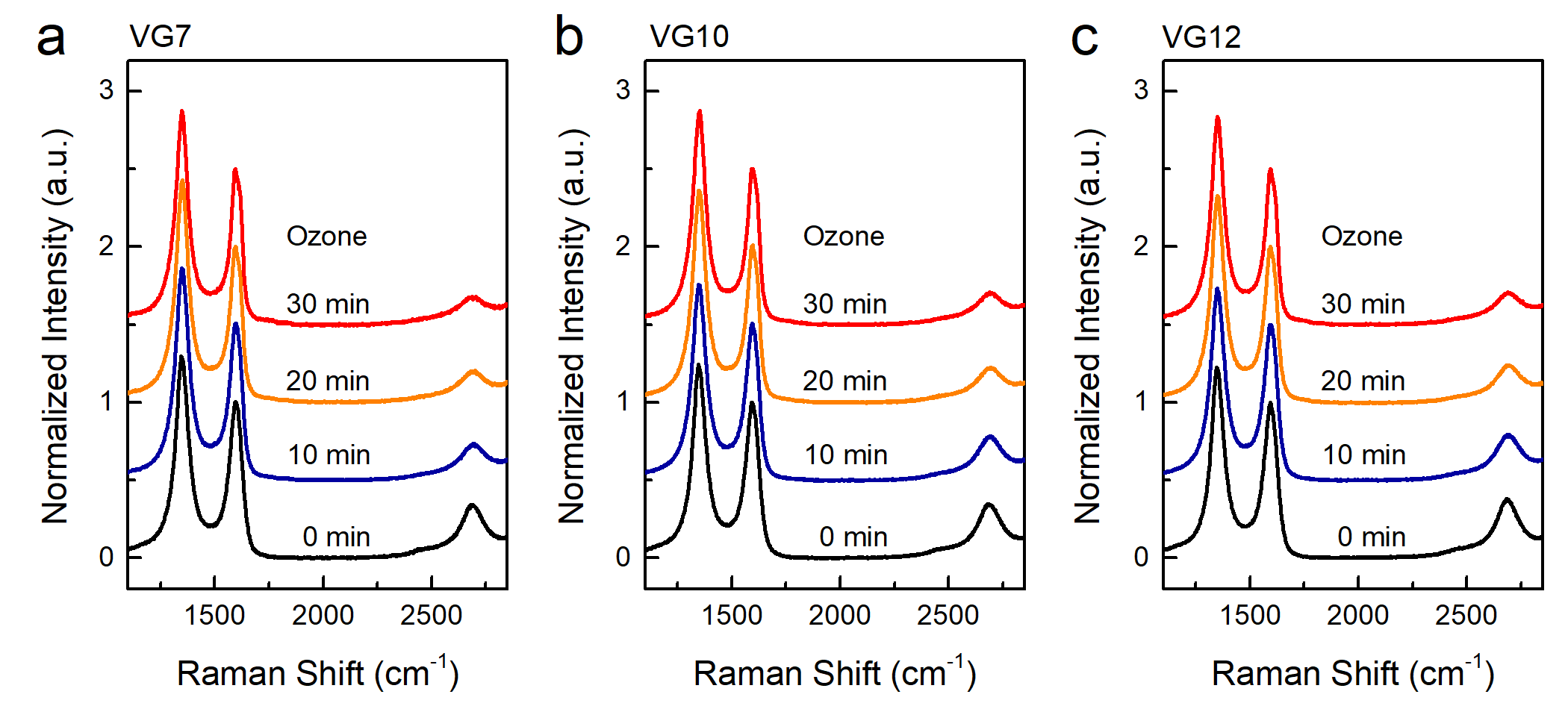


**Figure S4.** Raman spectra of a) VG7, b) VG10, and c) VG12. Peak ratio of d) D and e) 2D peaks to G peak. The D peak increases until the volume of VG vanishes by ozone treatment. The 2D peak of VG decreases continuously by ozone treatment.

S5. *J-V* characteristics for 30 min ozone treated VG7

Using the emission measurement set-up described in the main text, *J-V* characteristics were measured for VG7 (Figure S5). Without the ozone treatment, VG emits electrons poorly with ~1 % emission efficiency. The ozone treatment increases the emission current density, and the 30 min-ozone treated VG7 shows the highest efficiency of ~40 %. However, the lateral resistance of that sample is too large to define the gate voltage correctly, and the Fowler-Nordheim (FN) plot is not linear.


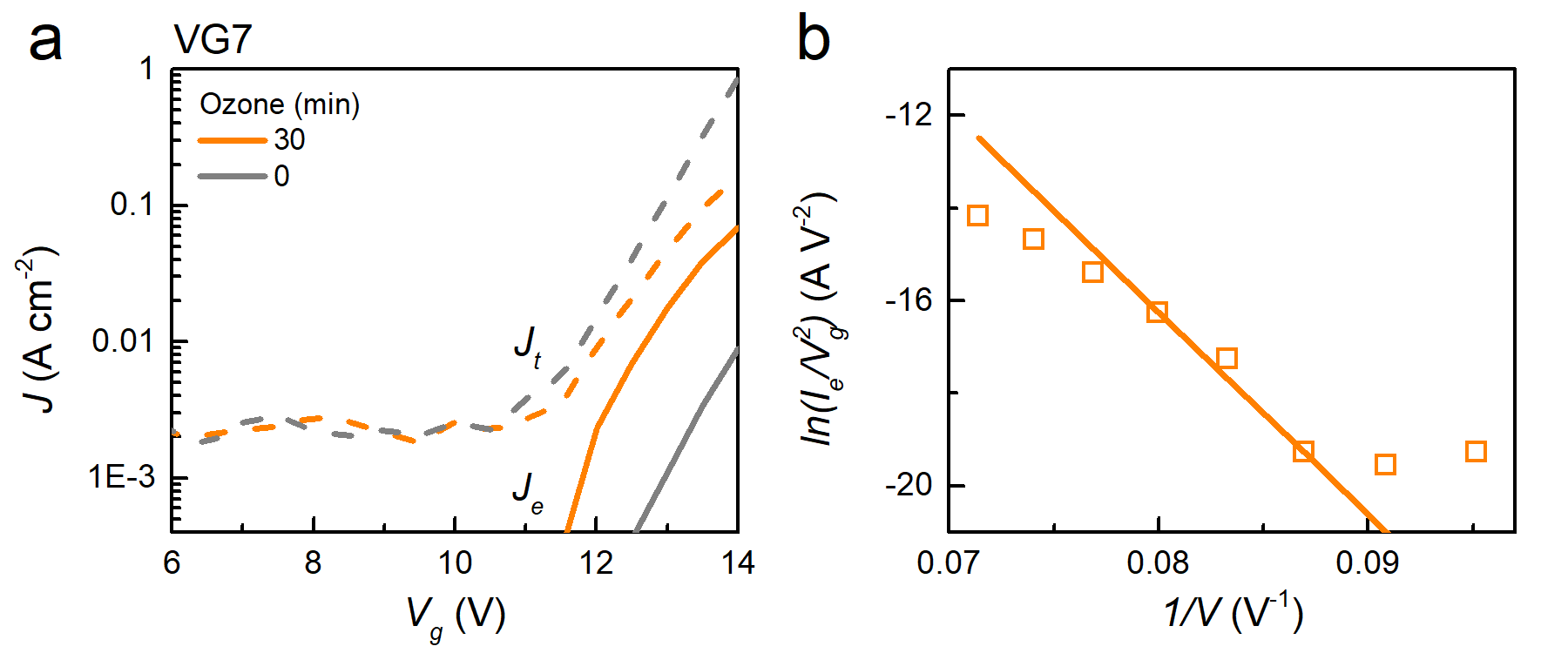


**Figure S5.** Electron emission properties for VG7. a) *J-V* curve of VG7 for 30 min ozone treatment and b) the corresponding FN plot for the emission.

S6. Electron emission cost

To determine a power efficiency, the electron emission cost was calculated by multiplying applied voltage (*V_g_*) to the total current (*I_t_*) per the emission current (*I_e_*). As shown in Figure S6, bare VG devices consume a huge power > 1 kW/A (*V_g_*=14 V), and the power cost drastically decreases for longer ozone treatment times. Table S1 presents comparisons of emission performances from other cold cathode emission devices.


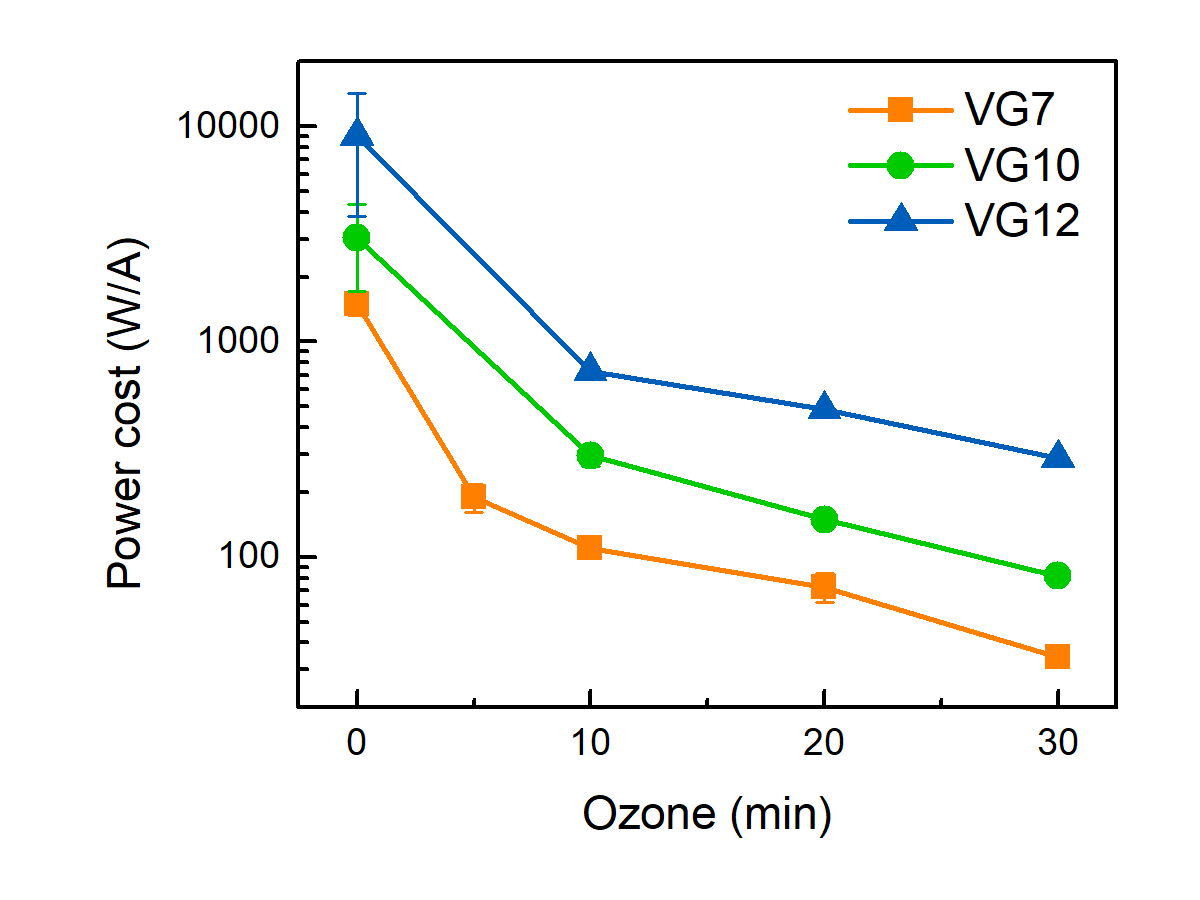


**Figure S6.** Power cost for electron emission as a function of ozone treatment time.

**Table S1.** Emission performances comparisons for cold cathode emission devices.

|  | Emission current (mA) | Emission current density (mA/cm2) | Electron emission cost (W/A) | Reference |
| --- | --- | --- | --- | --- |
| CNT | 3.0 | 0.04 | 320 | [6] |
| Si | 1.0 | 2.8 | 127 | [7] |
| GOS 380 cells | 6.0 | 67 | 57 | [8] |
| VG 5x5 array | 0.1 | 50 | 34 | This work |

S7. X-ray imaging system

Figure S7a presents a photograph of our high vacuum chamber for X-ray generation, with the schematics shown in Figure S7b. Emitted electrons from VG tunnelling cathode are accelerated by 60 kV and are directed to the 15 degree-off angled anode. After scattering occurred at the anode, the generated X-ray travels through the chest phantom to a detector panel with 115 cm of source-to-image distance (Figure S7c). The recorded X-ray image is presented in the main text, Figure 5e.


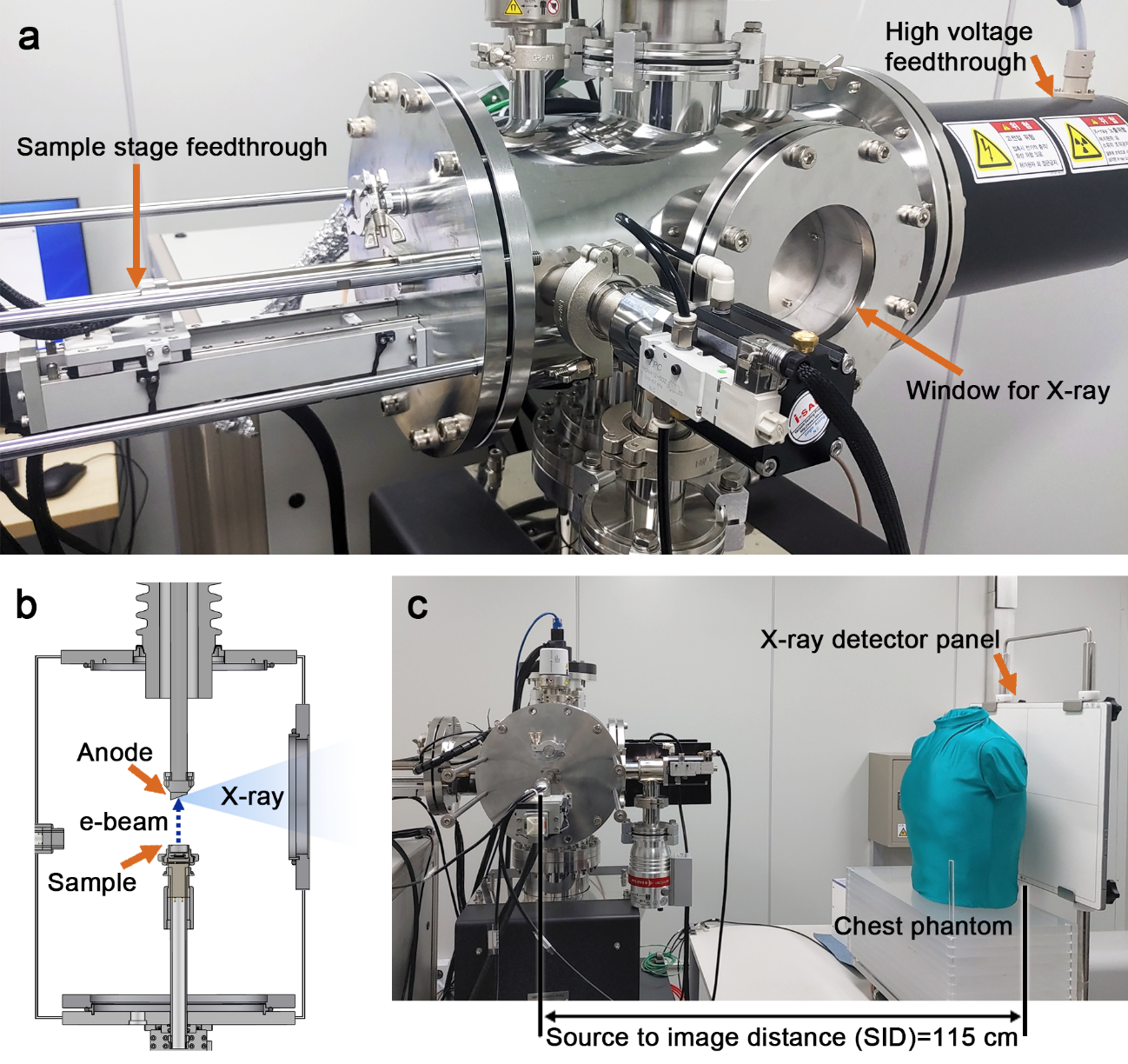


**Figure S7.** a) Photograph of X-ray generation chamber. b) Schematic illustration of X-ray generation. c) Photograph of X-ray imaging for the chest phantom.

**References**

[1] P. K. Katkar, A. N. Kadam, S. K. Jerng, S. H. Chun, S. W. Lee, *J. Alloys Compd.* **2023**, *968*, 171935.

[2] S. B. Roy, S.-K. Jerng, K. Akbar, J. H. Jeon, L. Truong, S.-H. Chun, M. J. Noh, J. Lee, Y.-H. Kim, *J. Mater. Chem. A* **2019**, *7*, 17046.

[3] K. Akbar, S. Hussain, L. Truong, S. B. Roy, J. H. Jeon, S.-K. Jerng, M. Kim, Y. Yi, J. Jung, S.-H. Chun, *ACS Appl. Mater. Interfaces* **2017**, *9*, 43674.

[4] S. B. Roy, L. Truong, J. H. Jeon, S. Lee, S.-K. Jerng, E. Jung, S. Chan Jun, S.-H. Chun, *ACS Appl. Energy Mater.* **2021**, *4*, 5697.

[5] H. R. Na, H. J. Lee, J. H. Jeon, H. J. Kim, S. K. Jerng, S. B. Roy, S. H. Chun, S. Lee, Y. J. Yun, *npj Flex. Electron.* **2022**, *6*, 1.

[6] N. Yamamoto, T. Morita, Y. Ohkawa, M. Nakano, I. Funaki, *J. Propuls. Power* **2019**, *35*, 490.

[7] A. A. Fomani, A. I. Akinwande, L. F. Velásquez-García, *J. Phys. Conf. Ser.* **2013**, *476*, 012014.

[8] R. Furuya, Y. Takao, M. Nagao, K. Murakami, *Acta Astronaut.* **2020**, *174*, 48.
